# Contribution of gut microbiota toward renal function in sepsis

**DOI:** 10.3389/fmicb.2022.985283

**Published:** 2022-09-06

**Authors:** Yaya Xu, Xiangmei Kong, Yueniu Zhu, Jiayue Xu, Haoyun Mao, Jiru Li, Jianhua Zhang, Xiaodong Zhu

**Affiliations:** ^1^Department of Pediatric Critical Care Medicine, Xinhua Hospital, Affiliated to the Medical School of Shanghai Jiao Tong University, Shanghai, China; ^2^Department of Pediatric Respiratory, Xinhua Hospital, Affiliated to the Medical School of Shanghai Jiao Tong University, Shanghai, China

**Keywords:** intestinal microbiology, augmented renal clearance, gut–kidney crosstalk, sepsis, gut micro flora

## Abstract

Sepsis most often involves the kidney and is one of the most common causes of acute kidney injury. The prevalence of septic acute kidney injury has increased significantly in recent years. The gut microbiota plays an important role in sepsis. It interacts with the kidney in a complex and multifactorial process, which is not fully understood. Sepsis may lead to gut microbiota alteration, orchestrate gut mucosal injury, and cause gut barrier failure, which further alters the host immunological and metabolic homeostasis. The pattern of gut microbiota alteration also varies with sepsis progression. Changes in intestinal microecology have double-edged effects on renal function, which also affects intestinal homeostasis. This review aimed to clarify the interaction between gut microbiota and renal function during the onset and progression of sepsis. The mechanism of gut–kidney crosstalk may provide potential insights for the development of novel therapeutic strategies for sepsis.

## Highlights

- Sepsis-associated acute kidney injury is a growing global health challenge, affected by changes in the composition and function of the gut microbiota.
- The presence of a “gut–kidney axis” facilitates bidirectional communication between gut microbiota and renal function and is involved in the development of sepsis.
- Loss of endothelial barrier integrity and dysbiosis of the gut microbiota are the primary pathophysiological alterations in sepsis, which play a critical role in sepsis-associated acute kidney injury in response to various pathological insults, with both positive and negative effects.
- The gut–kidney axis has the potential to be an important target in the treatment of sepsis-associated acute kidney injury.

## Introduction

Intestinal barrier dysfunction and gut microbiota disorders are important determinants of the occurrence and development of sepsis (Meng et al., 2017). As a vital component of the intestinal tract, the gut microbiota critically influences important physiological processes, such as biosynthesis, and metabolism (Yeh et al., 2016; Meng et al., 2017; Antushevich, 2020). The kidney is the organ that is most involved in sepsis. Studies have shown that patients with sepsis-associated acute kidney injury (SA-AKI) have a higher prevalence and more severe renal damage than that observed in those without SA-AKI (Zeng et al., 2014). Peng et al. (2014) reported that the incidence of SA-AKI was 11–31% and was as high as 41–78% in patients with septic shock. Paradoxically, patients with a normal serum creatinine (SCr) concentration may display augmented renal clearance (ARC) during the early stages of sepsis [i.e., the ability of kidneys to increase the glomerular filtration rate (GFR) in response to certain physiological or pathological stimuli] (Sharma et al., 2014). This counterintuitive and accelerated drug elimination was present in 39.5–56% of sepsis patients (Baptista et al., 2012; Carrié et al., 2018). Abundant evidence has indicated that the gut microbiota is involved in the regulation of renal function in sepsis patients (Miller et al., 2021). The core theoretical view of the “gut–kidney axis” is that during the progression of kidney damage, there are intestinal microecosystem disorders, including a decrease in beneficial bacteria and an increase in pathogenic bacteria that can produce urotoxin, leading to the accumulation of intestinal urotoxin in the blood. These toxins cannot be cleared by the damaged kidneys in time, thus exacerbating kidney damage and eventually forming a vicious circle between the intestines and kidneys (Nakade et al., 2018; Wu et al., 2020). The intestinal epithelial barrier in sepsis patients is compromised, such that intestinal urotoxin and conditional pathogenic bacteria are displaced and enter the blood circulation, activating the intestinal mucosal immune system and inducing systemic inflammatory response syndrome (SIRS) (Gao et al., 2015; Tan R. Z. et al., 2020). Some studies have suggested that a diverse gut microbiota can delay kidney aging and improve renal function (Nie et al., 2022). Deciphering the interaction between gut microbiota and renal function in sepsis patients, as well as its underlying mechanisms, is a major public health challenge in the development of new prevention or treatment strategies. In this review, we focused on studies exploring the effects of altered gut microbiota on renal function in sepsis patients.

## Gut microbiota in sepsis

Most gut microbial species belong to four major phyla: Firmicutes, Actinobacteria, Proteobacteria, and Bacteroidetes. Gut microbial species in the phyla Bacteroidetes and Firmicutes account for more than 90% of all bacteria (Ramakrishna, 2013; Chen, 2016; Lankelma et al., 2017b; Akash et al., 2019; Firmino et al., 2020).

### Dysbiosis of gut microbiota in sepsis patients

**FIGURE 1:**
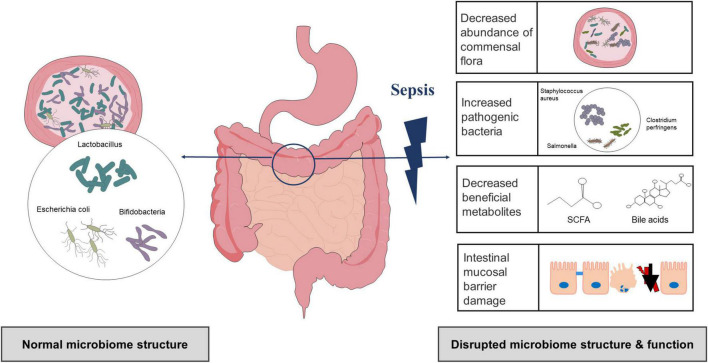
Gut microbiota alterations in sepsis.

Compared with the healthy control group, there were differences in the number and distribution of bacteria as well as in the microbiota structure of the sepsis group (Yang et al., 2021). Several studies have shown that the ecological and functional microenvironment of the gut changes when sepsis occurs (Figure 1; Bassetti et al., 2020; Kullberg et al., 2021; Sun et al., 2022). First, gut microbiota diversity and richness are substantially decreased in sepsis. Using lipopolysaccharide (LPS)-induced septic shock and cecal ligation and puncture (CLP)-induced polymicrobial septic models, a study found that non-sepsis samples had a distinct bacterial diversity that was significantly enriched (i.e., Chao1 index, 2151.26 ± 230.87 *vs.* 1800.81 ± 325.82) and more diverse (i.e., Shannon index, 6.27 ± 0.39 *vs.* 5.29 ± 0.72) compared to that of sepsis (Chen et al., 2019). In another study, the gut microbiota of the healthy controls had a significantly higher Shannon index than that of the sepsis group (3.49 ± 0.45 *vs*. 2.98 ± 0.68) (Zhu et al., 2018). Second, there are relative changes in the community structure and composition of the gut microbiota during sepsis (Dickson, 2016). The most significant change in the gut microbiota composition in sepsis patients is the decrease in protective symbiotic flora, particularly obligate anaerobic bacteria and lactobacilli, and the abundance of pathogenic bacteria such as *Enterococcus* and *Pseudomonas* increases and can dominate the gut flora (Dickson, 2016; Lankelma et al., 2017b; Wan et al., 2018). In a recent prospective study involving intensive care unit (ICU) sepsis patients at admission, a relative high abundance of Proteobacteria was observed in approximately one-third of patients (healthy controls *vs.* ICU patients, *P* = 0.02), and the proportion of Gram-positive and Gram-negative bacteria also increased (Lankelma et al., 2017b). Another study also confirmed a higher abundance of Proteobacteria in sepsis patients (mean relative proportion, 23.71 *vs.* 3.53% in septic shock patients *vs.* healthy controls) (Wan et al., 2018). Shimizu et al. reported changes over time in the most abundant phyla, Bacteroidetes, and Firmicutes, in patients with SIRS (Ojima et al., 2016). At the same time, patients who presented with extremes have poor outcomes, including increased in-hospital mortality (Ojima et al., 2016). Furthermore, changes in the gut microbiota appear to result in chain reactions. An increase in pathogenic bacteria can degrade and penetrate the protective mucus layer, followed by adhesion and colonization of intestinal epithelial cells, disruption of the intestinal mucosal barrier, and exacerbation of infection (Paone and Cani, 2020). Animal experiments have shown that a decrease in beneficial intestinal bacteria in mouse models of sepsis could exacerbate the intestinal colonization and virulence of *Pseudomonas aeruginosa* (*P. aeruginosa*) (Christofi et al., 2019). Notably, antibiotic therapy, the main therapeutic measure for sepsis, can also lead to changes in the gut microbiota. Christofi et al. administered a regime containing three broad-spectrum antibiotics in mice and found that *Escherichia coli (E. coli)* was detected in a mouse model of sepsis without antibiotic treatment, but not in antibiotic-treated mice, which become susceptible to opportunistic infection. They also point out that antibiotic treatment induced dysbiosis, which is exemplified by eradication of *E. coli* as well as reduction of all commonly prevalent phyla in the gut to undetectable levels (Christofi et al., 2019). This observation is similar to the results of other human studies (Lankelma et al., 2017a; Du et al., 2021). Many broad-spectrum antibiotics inhibit or kill dominant flora, leading to increased colonization of conditioned pathogenic bacteria and fungi, and ultimately resulting in opportunistic or secondary infections and exacerbation of intestinal microbiome imbalances (Song and Kim, 2019). Several other studies have suggested that the use of broad-spectrum antibiotics increases the risk of subsequent *Clostridium difficile* (*C. difficile*) infections (Deshpande et al., 2015).

### Alterations in the gut ecological and functional microenvironment in different stages of sepsis

Hayakawa et al. (2011) found that the gut microbiota changed significantly within the first 6 h of critical illness, mainly manifested by a significant decrease in the protective flora, especially obligate anaerobes, whereas harmful bacteria, such as *Enterococcus faecalis* and *P. aeruginosa*, increased, accompanied by a decrease in the three main short-chain fatty acids (SCFAs), including butyrate, propionate, and acetate. Zhu et al. (2018) indicated that the gut microbiota composition changed gradually over time. We summarized the most relevant clinical studies published to date on sepsis patients that correlated with the gut microbiota (Supplementary Table 1). A flowchart of the literature search and screening process was shown in Supplementary Figure 1.

Within 48 h following the confirmation of sepsis, the abundance of the gut microbiota and the bacterial diversity in sepsis patients were reduced, as compared with those in healthy controls, and this phenomenon could persist for approximately 1°week. Moreover, treatment with antibiotics or probiotics could affect the outcomes. In seven clinical studies, the dominant gut microbiota was different in sepsis patients, mainly Firmicutes and Proteobacteria (Shimizu et al., 2018; Liu et al., 2019, 2020, 2021; Stadlbauer et al., 2019; Du et al., 2021; Reyman et al., 2022). Notably, Niu and Chen, 2021 found that the composition and functional components of the gut microbiota in sepsis patients exhibited a robust circadian rhythm. Current evidence suggested an increase in the abundance of Firmicutes during the eating phase and an increase in Actinobacteria and Proteobacteria during the fasting phase (Parkar et al., 2019). Based on the circadian interactions between the host and microbiome, strict regulation between the gut microbiota and immune system will aid in the development of more precise therapies for treating sepsis. Additionally, significant individual differences have been noted among these studies (Shimizu et al., 2018; Stadlbauer et al., 2019; Liu et al., 2020, 2021; Du et al., 2021; Reyman et al., 2022). Therefore, changes in the gut microbiota during sepsis cannot be generalized. The results of some studies require further verification by clinical randomized controlled trials; nevertheless, a few trials investigating the gut microbiota in sepsis patients (e.g., NCT02469571 and TCTR20191017004) are currently underway.

## Gut–kidney crosstalk in sepsis

Changes in the gut microbiota in sepsis not only are diverse but also have various effects on the body.

### Gut microbiota has deleterious effects on the kidneys

**FIGURE 2:**
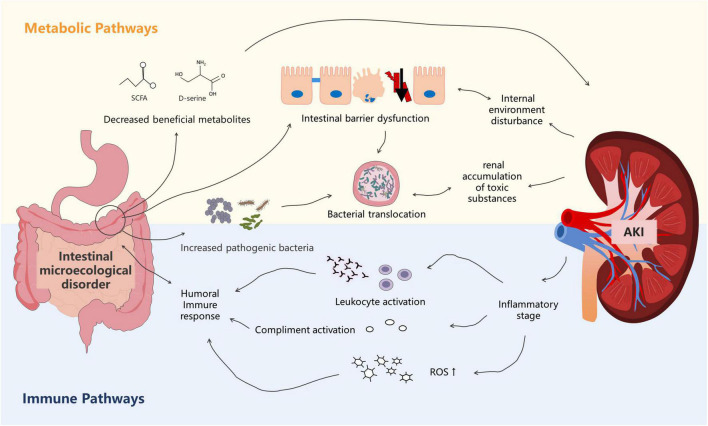
The metabolism-dependent and immune pathways of the gut–kidney axis in sepsis. Dysbiosis of the gut microbiota is associated with altered metabolic pathways that cause accumulation of uremic toxins, leading to kidney injury. Kidney injury, in turn, affects the gut microecology. In the immune pathway, products derived from bacterial members of the gut microbiota evoke immune signaling pathways in the host, leading to systemic inflammatory syndrome. Kidney injury also affects immunity.

Meijers and Evenepoel (2011) proposed the concept of “gut–kidney axis” in 2011. Subsequently, Pahl and Vaziri, 2015 refined this theory. The foundation of this theory is that kidney injury can lead to gut microbiota disorders and damage the intestinal epithelial barrier function. Intestinal microecological imbalance can also produce metabolic toxins, aggravating kidney injury. With the increasing availability of genomics, the “microbiota-toxin-barrier-inflammation” event chain of the gut–kidney axis has been elucidated (Figure 2).

First, sepsis itself can affect the abundance and composition of the gut microbiota, which can subsequently promote the occurrence and development of acute kidney injury (AKI) (Emal et al., 2017; Yuan et al., 2020). Nakade et al. (2018) found that fecal transplantation from normal mice attenuated renal pathology in germ-free C57BL/6 mice, suggesting that the gut microbiota in normal mice may produce molecules that protect the kidneys. Recent studies have indicated that, among the bacterial metabolites, SCFAs play a protective role in AKI (Andrade-Oliveira et al., 2015). In addition to SCFAs, gut microbiota–derived D-serine has also been suggested to improve kidney injury (Sasabe et al., 2016; Nakade et al., 2018). Paradoxically, Emal et al. (2017) found that gut microbiota depletion with broad-spectrum antibiotics protected against renal ischemia–reperfusion (I/R) injury. Mechanistically, Emal et al. (2017) reported that microbiota depletion significantly attenuated renal damage by reducing the maturation status of F4/80 renal-resident macrophages and bone marrow monocytes. These two different mechanisms suggest that the gut microbiota exerts multiple effects on the kidneys. Changes in the gut microbiota composition also play an important role in renal function. Gut microbiota alteration leads to the release of inflammatory mediators that pass into the circulation, thereby affecting the renal function. Druml (2014) suggested that the release of cytokines and inflammatory mediators increased oxidative stress, activation of various immune cells, neutrophil extravasation, and generalized kidney injury. Using a model of endotoxin-induced sepsis, Yu et al. (2018) showed that IL-1β, TNF-α, MCP-1, and NOS-2 levels were significantly increased in kidney tissues. Li et al. (2019) also showed that gut-derived endotoxin, resulting from increased intestinal permeability after severe renal I/R, subsequently amplified the intrarenal inflammatory response by activating renal TLR4 signaling. Furthermore, gut microbiota alteration leads to an increase in the oxidative stress level in AKI and exacerbates the ischemic injury. Xie et al. (2020) described that a significant increase in *Helicobacter* in the gut microbiota of mice might directly or indirectly affect the oxidative stress level through serum biochemical indicators. Moreover, *Clostridium* was negatively correlated with superoxide dismutase and glutathione peroxidase but was positively correlated with malondialdehyde.

Second, sepsis can disrupt the intestinal barrier, exacerbating the impact of the gut microbiota on the kidneys. Many studies have shown that sepsis causes changes in the integral and peripheral membrane proteins (i.e., myosin) that make up tight junctions, leading to significant increases in intestinal permeability (Meng et al., 2017; Paone and Cani, 2020). Lorentz et al. (2017) found that myosin light chain kinase (MLCK) in mouse models of sepsis phosphorylated myosin regulatory light chains, impairing the intestinal barrier function and increasing permeability. The survival rate of MLCK-knockout mice was significantly higher than that of the control group (95 *vs.* 24%, respectively, *P* < 0.0001), and the rates of kidney injury were also lower. The intestinal chemical barrier is also disrupted in sepsis patients owing to changes in the gut microbiota composition (Paone and Cani, 2020). Although the precise mechanism remains unknown, Johansson et al. (2015) suggested that it may be related to changes in glycosyltransferase expression levels and that O-glycan patterns dynamically change with changes in the gut microbiota. Using a model of Goodpasture disease that resulted in nephrotoxic nephritis, Pagan et al. (2018) found that extracellular glycosyltransferases likely influence IgG Fc glycoforms and could attenuate inflammation. Several studies have shown that damage to the intestinal mucosal barrier may stimulate pro-inflammatory events, exacerbating the displacement of the microflora, which can cause kidney damage (Druml, 2014; Capaldo et al., 2017; Li et al., 2019).

Acute kidney injury (AKI) also affects the gut microbiota. A recent study using a model of AKI reported a decreased abundance of *Bifidobacterium* and an increased abundance of *Lactobacillus*, *Clostridium*, and *Ruminococcus* after I/R injury (Nakade et al., 2018). The authors suggested that this finding might result from the protective effect of the gut microbiota against tubular injury in this mouse model of AKI. Further studies suggested that the protective effect may be related to T-helper 17 (Th17) cells (Krebs et al., 2016). Krebs et al. (2016) found that Th17 cells egressed from the gut in an S1P-receptor-1-dependent fashion and subsequently migrated to the kidney *via* the CCL20/CCR6 axis, and depletion of intestinal Th17 cells in germ-free and antibiotic-treated mice ameliorated renal disease. However, this self-regulatory mechanism has a limited effect. The inflammatory response triggered by AKI and the high volume load altered vascular permeability, leading to vascular leakage and angioedema, eventually disrupting intestinal epithelial tight junctions, increasing intestinal epithelial cell apoptosis, and suppressing epithelial turnover (Gong et al., 2019). Takeda et al. (2021) found that ileal villous epithelial cells in a rat model of AKI were shed, and the integrity of the intrinsic layer was impaired. Persistent impairment of the permeability and integrity of the intestinal epithelial barrier led to spillage of bacteria, endotoxins, and/or macromolecular substances, exacerbating sepsis (Hudson et al., 2017; Nakade et al., 2018). Moreover, Ranganathan et al. (2010) indicated that the metabolites produced by this altered gut microbiota resulted in the accumulation of uremic toxins, such as indoxyl sulfate (IS) and p-cresyl sulfate, causing kidney injury by contributing to the inflammatory infiltrate and tubular atrophy.

From an immunological perspective, the cytokines released by the kidneys can have an important effect on the intestines through the circulation (Hato and Dagher, 2015). Using a mouse model of LPS-induced endotoxemia, Quan et al. (2020) showed that B cell-activating factor levels were significantly upregulated in the kidneys, whereas serum IL-1β, IL-6, and TNF-α levels were significantly increased. Furthermore, they revealed that inflammatory mediator in the blood circulation influenced the gut microbiome by increasing intestinal hyperpermeability (Quan et al., 2020). Further studies have indicated that TLR4 activation in SA-AKI could result in the release of cytokines and chemokines as well as leukocyte infiltration, which lead to endothelial and renal tubular dysfunction and further contribute to this inflammatory state, causing multiple organ dysfunctions syndrome (Anderberg et al., 2017). Wang L. et al. (2016) found that the activation of the TLR4 receptor increased the release of endotoxins from the gut microbiota, leading to SIRS and aggravated AKI.

### Unraveling the contribution of the gut microbiota to the development of augmented renal clearance

**FIGURE 3:**
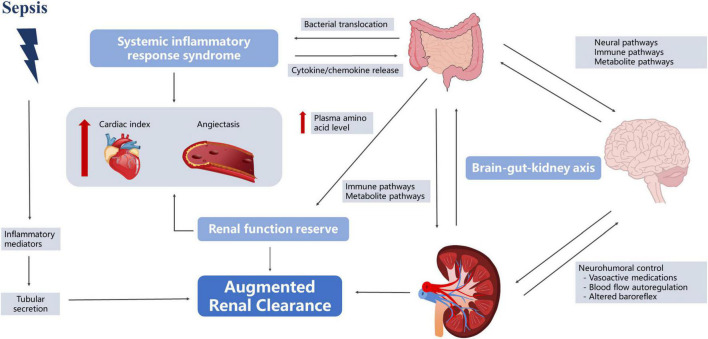
Roles of the gut microbiota in kidney protection.

In 2010, Udy et al. (2010) found that patients with severe disease often experienced hyperfunction in the early stages of the disease, and some began to develop kidney damage as the disease progressed. Grootaert et al. (2012) observed that 30% of severely ill patients had at least one episode of ARC, lasting for 5–7 days on average. Most studies defined ARC as a creatinine clearance rate of ≥ 130 mL/min/1.73 m^2^ (De Waele et al., 2015; Mahmoud and Shen, 2017; Bilbao-Meseguer et al., 2018). However, the understanding of the fundamental mechanisms of ARC is incomplete. Currently, three main mechanisms of ARC are widely accepted—namely, SIRS, renal function reserve (RFR), and brain–kidney crosstalk (Cook and Hatton-Kolpek, 2019; Luo et al., 2021). Interestingly, all of them appear to be linked to the gut microbiota (Figure 3).

#### The theory of systemic inflammatory response syndrome

In the early stages of sepsis, the organism is in a state of hypermetabolism, accompanied by the release of pro-inflammatory mediators, which may reduce vascular resistance and increase cardiac output, leading to increased renal blood flow and changes in glomerular filtration (Langenberg et al., 2005; Trof et al., 2010). Gut microbiota plays an important role in this process. First of all, in sepsis, intestinal permeability increases, bacteria and toxins originally present in the intestinal lumen pass through the intestinal wall, direct venous invasion to distant organs with involvement of lymph nodes and proliferate in large quantities, subsequently causing constant generation of inflammatory cytokines and leading to the “waterfall” cascading effects (Vaishnavi, 2013; Paone and Cani, 2020). Second, gut microbiota affects vascular resistance. Traykova et al. (2017) reported that when bacterial translocation occurred, the number of bacterial species and the total bacterial DNA were positively correlated with the serum nitric oxide level [odds ratio (OR): 0.64, 95% confidence interval (CI): 0.247–0.852, *P* = 0.0041 and OR: 0.62, 95% CI: 0.216–0.842, *P* = 0.0062, respectively] and were inversely correlated with systemic vascular resistance (OR: −0.593, 95% CI: −0.83 to −0.175, *P* = 0.0095 and OR: −0.621, 95% CI: −0.843 to −0.218, *P* = 0.0060, respectively). Huang et al. (2021) also found that fecal microbiota transplantation (FMT) restored the normal gut microbiota composition and downregulated phosphorylated endothelial nitric oxide synthase, leading to improved vasodilation. Therefore, we speculated that the gut microbiota in sepsis patients may promote increased renal blood flow by participating in the SIRS response, leading to ARC. However, relevant research has not directly reached this conclusion. Although studies have shown that the gut microbiota affected nitric oxide expression, these reports experimented with cirrhosis, and it is unclear whether the underlying disease affected this process (Traykova et al., 2017; Huang et al., 2021).

#### The theory of renal function reserve

In the resting state, the kidneys work with basic abilities, and can make corresponding adjustments according to physiological stimuli, increasing to a certain high-level state (Thomas et al., 1994). The earliest RFR elaboration can be traced back to 1983. Bosch et al. (1983) found that normal subjects had a significant increase in GFR after acute protein loading (1.0–1.2 g/kg). RFR is also present in sepsis patients, and Belcher et al. (2011) found that in the early stage of sepsis, the increase in SCr in patients was not obvious due to the strong reserve function of the kidneys. Langenberg reviewed experimental animal and human studies of SA-AKI and found that 62% of animals showed a decrease in renal blood flow (RBF), while the remaining 38% exhibited no change or increase in RBF (Langenberg et al., 2005). Maiden et al. (2016) monitored sheep models of sepsis and found that renal oxygen consumption did not change with disease severity. However, the stimulation conditions for RFR in sepsis patients remain unclear, and multiple studies have suggested that protein intake may be involved, and may increase GFR through vasodilation and increased RBF (Bosch et al., 1983; Sharma et al., 2014; Ronco et al., 2017). Interestingly, this phenomenon also seems inextricably linked to gut microbiota. First, dysbiosis of the gut microbiota after a high-protein diet triggers an inflammatory response. Rong et al. found that bacteria that contribute to protein fermentation in a high-protein diet were significantly higher than those in the control group (Tan R. et al., 2020). The proliferation of pathogenic microbiota leads to increased intestinal permeability, and a study also found that the levels of serum inflammatory markers in mice on high-protein diets and the water level of serum diamine oxidase, a classical permeability marker, demonstrated that high-protein diets could cause increased intestinal permeability and the occurrence of intestinal inflammation (Tan R. et al., 2020). Therefore, we speculate that a high-protein diet may result in increased RBF through the inflammatory response pathway, which in turn causes an increase in the GFR. Second, the amino acid metabolites of the intestinal flora increased in the high-protein diet group, and the intestinal mucosa was damaged. SCFAs, hydrogen sulfide, indole compounds, histamine, and serotonin are amino acid-derived bacterial metabolites that play an important role in stimulating the immune response and maintaining the integrity of the intestinal epithelial barrier. Andriamihaja et al. (2010) showed that a high-protein diet significantly reduced the height of the absorptive colonic epithelial cell brush border in mice while reducing cellular energy production. Toden et al. (2005) showed that a high-protein diet could cause an increase in cresol levels in bacterial-derived genotoxic metabolites, which in turn induced DNA damage in colon cells. Wu et al. (2001) found that plasma amino acid levels gradually increased at 6 h after sepsis in a rat model intraperitoneally injected with endotoxins and reached a peak after 24 h. Additional studies have found that elevation of plasma amino acid levels increased proximal tubular reabsorption and endothelium-derived relaxing factors, leading to blood vessel dilation and RBF increase (Sharma et al., 2014). RFR reserves are limited and largely depend on complete renal function. When more than 50% of the renal units are destroyed, renal functional reserves are lost and SCr values tend to increase (Ronco et al., 2017).

#### The theory of brain–kidney crosstalk

Dias et al. (2015) found that intracranial pressure, cerebral perfusion pressure, and cerebrovascular reactivity pressure index were significantly associated with ARC in patients with traumatic brain injury, suggesting the presence of a “brain–kidney crosstalk.” Yang et al. (2018) further proposed the concept of the brain–gut–kidney axis. The gut microbiota communicates with the brain through three parallel and interacting pathways, including the neural, immune, and metabolite pathways (Yu and Zhao, 2021). Evidence suggests that the vagus nerve can sense microbial signals in forms of bacterial metabolites or be affected by the microbiota-mediated regulation of enterocrine and intestinal epithelial cells (Margolis et al., 2021). Nerve fluid axis activation further affects renal function by regulating RBF (Bouglé and Duranteau, 2011). Notably, changes in the limbic system of sepsis patients regulated intestinal immunity through the autonomic nervous system, acetylcholine anti-inflammatory pathways, hypothalamic–pituitary–adrenal axis, and neuropeptides (Ye et al., 2019). Ben-Shaanan et al. (2016) used direct neuronal manipulation to establish a causal relationship between the brain’s reward system and immunity and found that activation of the reward system increased the primary antibacterial immune response and that after pathogen re-exposure. Increased paracellular permeability during brain injury may contribute to gut microbiota translocation and promote systemic inflammatory responses (Li X. J. et al., 2020). Inflammation induced by gut pathogens in turn increased the risk of brain damage (Franceschi et al., 2019).

Metabolites produced by the gut microbiota can act as chemical signals that directly or indirectly regulate homeostasis of the central nervous system. Studies have shown that D-serine can be synthesized and secreted by glial cells and act as a co-agonist of the extrasynaptic *N*-methyl-*D*-aspartate subtype of glutamate receptors, which are involved in neurodegenerative disorders and cell death (Holeček, 2022). Nakade et al. (2018) showed that increased D-serine secretion improved renal function.

## Gut–kidney axis-based treatment for sepsis

Based on the aforementioned mechanisms of enterorenal interaction in sepsis, some studies have suggested that the gut microbiota is the target of treatment for SA-AKI, which can be divided into the following three categories according to its mechanism: (i) probiotic supplementation or gut microbiota metabolite supplementation/inhibition, (ii) selective digestive tract decontamination (SDD), and (iii) microbial replacement therapies (Yuan et al., 2020; Kullberg et al., 2021; Zhu et al., 2021).

### Supplementation or inhibition of gut microbiota metabolites

Fecal microbiota transplantation (FMT) carries certain risks, and direct supplementation with gut microbiota-related metabolites to improve renal function is a hot topic. Multiple studies have reported that treatment with SCFAs reduced kidney injury in various animal models (Machado et al., 2012; Sun et al., 2013; Andrade-Oliveira et al., 2015). SCFAs protect the kidneys through mechanisms, including anti-inflammatory effects, immune regulation, and intestinal barrier reparation (Hayakawa et al., 2011; Andrade-Oliveira et al., 2015). Therefore, SCFAs are expected to become a new type of drug to improve renal function. However, the amount and timing of SCFAs supplementation are still matters of debate. The effective concentration of SCFAs for renoprotective effects in the aforementioned studies is also markedly higher than their physiological concentration, and the safety of their clinical application remains to be demonstrated. We believe that giving full consideration to the positive effects of gut microbiota on renal function can also improve kidney damage. Si et al. (2013) found that adrenocorticosteroid gel could prevent tumor necrosis factor-induced damage to the renal tubular cells in rats and confirmed that the use of the brain–gut–kidney axis could also exert a protective effect against kidney damage. In addition, the suppression of harmful metabolites is the focus of improving renal function. Protein can be transformed by gut microbiota into a variety of metabolites including p-cresol sulfate and IS, which, as uremic toxins, can cause renal damage by promoting mononuclear/macrophage infiltration (Ranganathan et al., 2010; Wu et al., 2020). Currently, the best treatment strategy against toxins produced by gut microbiota metabolism is to change the gut microenvironment, including dietary adjustment, the use of probiotics, and targeted intervention in the gut (Skye and Hazen, 2016). Animal studies have shown that administration of AST-120 resulted in the reduction of plasma endotoxin and inflammatory cytokines, chemokines, and adhesion molecules, thereby attenuating systemic inflammation (Vaziri et al., 2013). Hatakeyama et al. (2012) found that the use of AST-120 in patients with chronic kidney disease (CKD) could reduce IS levels in blood and urine and delay the time for CKD patients to enter dialysis. Of interest, in 2016, Devlin et al. (2016) knocked out tryptophanases in mice to control IS production, a true case of targeting gut microbiota for disease treatment. This finding suggested that the host IS concentration can be controlled by targeting the gut microbiota, which may be a possible strategy to treat kidney injury.

### Probiotic supplementation

A prospective study conducted on patients receiving oral probiotics [90 billion colony forming units (CFUs)/day] reported decreases in urea nitrogen (*n* = 29, 63%), SCr (*n* = 20, 43%), and uric acid levels (*n* = 15, 33%) (Ranganathan et al., 2010). Zhu et al. (2021) showed that oral administration of *Lactobacillus casei* Zhang could alleviate kidney injury and that this probiotic as a preventive intervention could improve gut microbiota disorder, intestinal inflammation, and intestinal mucosal barrier injury caused by renal I/R injury. Mechanistically, a number of studies showed that probiotics could improve the immune function of sepsis patients by effectively reducing the inflammatory response (Wu et al., 2017; Bassetti et al., 2020; Zhu et al., 2021). However, some researchers have questioned the benefits of probiotics in SA-AKI treatment. Two reviews in 2019 noted that current clinical trials of probiotics have not been highly consistent, and the quality of evidence is often low (Abbasi, 2019; The Lancet Gastroenterology Hepatology, 2019). Zmora et al. (2018) studied 25 healthy volunteers who took probiotics for 2°months and found that whether probiotics could take root in the gut was entirely different in each participant. More targeted use of probiotics in the future, particularly based on an individual’s specific microbiome composition, may benefit SA-AKI patients.

### Antibiotics-based selective digestive tract decontamination

While the early use of antibiotics for sepsis patients has been a diagnosis and treatment consensus, the impact of antibiotics on gut microbiota cannot be ignored. Emal et al. used broad-spectrum antibiotics (1 g/L ampicillin, 1 g/L metronidazole, 1 g/L neomycin, and 0.5 g/L vancomycin) for SDD. Reduced gut microbiota can affect the maturation status of macrophages/monocytes and the release of chemokines, thus ameliorating renal I/R injury (Emal et al., 2017). In contrast, Lee et al. (2021) found that some antibiotics, such as ciprofloxacin, increased the secretion of Shiga toxin 2 (Stx2), by enhancing the expression of Stx2, thereby increasing the risk of hemolytic uremic syndrome. However, these two studies examined different subjects, antibiotics, and time frames. In addition, Lee et al. found that gentamicin is more efficient in reducing O157 infection and STX2-induced renal cell damage compared to ciprofloxacin, indicating that the effects of different antibiotic classes on gut microbiota are largely unknown. It is important to note that, according to the SIRS theory, the effects of early inflammatory responses on the kidneys are not all unfavorable, and it is debatable whether active use of broad-spectrum antibiotics to deplete the intestinal flora increases the risk of kidney damage (Langenberg et al., 2005; Trof et al., 2010). Ng et al. tracked microbiota dynamics with high temporal and taxonomic resolution during antibiotic treatment in a controlled murine system, isolating for variables such as diet, treatment history, and housing co-inhabitants, and found transient dominance of resistant *Bacteroides* and sustained loss in α-diversity during antibiotic treatment. Initial antibiotic treatment can condition the Bacteroides response of a human microbiota for subsequent treatments, suggesting the importance of FMT and targeted microbial treatments in restoring gut microbiota (Ng et al., 2019).

### Microbial replacement therapies: Benefits and risks

Microbial replacement therapies, particularly FMT, refer to the process of transplanting the gut microbiota from healthy donors to patients in order to reconstruct the normal intestinal microecology. FMT plays an important protective role through anti-inflammatory and antioxidant mechanisms, as well as the supplementation of intestinal probiotic metabolite production (Xiao et al., 2020). Nakade et al. (2018) showed that FMT from normal mice attenuated renal I/R injury by increasing the D-serine levels. Assimakopoulos et al. reported that FMT had a positive effect on intestinal barrier integrity by inhibiting the nuclear factor kappa-light-chain enhancer of activated B cells and increasing the expression of occludin (56 ± 15%) and claudin-1 (84 ± 7%). Moreover, FMT could restore immune ecological regulation by increasing the number of Paneth cells and inhibiting the release of inflammatory factors (Assimakopoulos et al., 2021). Li B. et al. (2020) showed that FMT could restore the gut microbiota, increasing the production of beneficial bacteria and SCFAs, restraining harmful bacteria, and inhibiting the activation of the TGF-β1/Smad/ERK signaling pathway. Nonetheless, thus far, no clinical studies have shown the effects of FMT on renal function. As such, this research direction may be further explored in the future. Moreover, long-term safety trials on FMT are lacking, with some studies suggesting that FMT is not always beneficial (Wang S. et al., 2016). Emal et al. (2017) reported that FMT exacerbated kidney damage in mice treated with antibiotics, suggesting that this result might be related to the inflammatory response produced by the gut microbiota. Wang et al. found that, among 7,562 studies in the literature, 78 reported FMT-related adverse events, of which serious adverse events included death (3.5%), inflammatory bowel disease recurrence (0.6%), and *C. difficile* infection (0.9%). The occurrence of FMT-related adverse events has been shown to be directly related to donor selection and closely related to transplantation methods (Li, 2020). Notably, the timing and composition of FMT are also topics of debate. Li et al. suggested that the future development direction should focus on personalized FMT based on donor–recipient precision microbiota analysis so that the current practice of whole fecal transplantation progresses to precision transplantation treatment using specific gut microbiota (Li, 2020).

## Concluding remarks

The interaction between the gut and kidney remains an interesting and unsolved mystery. During sepsis, the gut microbiota and intestinal mucosal barrier are destroyed, and the effects of alteration on renal function are double-edged. Renal function, in turn, affects the gut microbiota. Currently, the role of the gut–kidney axis in the occurrence and development of sepsis is poorly understood. Therefore, large-scale, well-controlled translation and preclinical studies are needed to evaluate the changes in the gut–kidney axis during sepsis at different time windows. SA-AKI treatment strategies based on the gut microbiota have been studied. In this regard, rational therapies, which theoretically give full consideration to the positive effects of the gut microbiota on the kidney while restoring intestinal integrity and maintaining the homeostasis of the gut microbiota, represent an exciting way to fight critically ill diseases.

## Author contributions

YX, XK, and YZ designed the systematic review. JX, HM, and JL conducted the literature search and assessed data quality. YX and XK drafted the manuscript and figures. JZ and XZ critically revised the review. All authors approved the final submitted version for publication.

## Supplementary material

Supplementary Figure 1 Flow chart of literature selection process. Studies were included if they met the following criteria: (i) it was a randomized controlled trial targeting the gut microbiota of sepsis patients; (ii) all subjects were free from the original heart, brain, endocrine, and other basic diseases; (iii) species identification was performed by DNA sequencing of 16S rDNA; (iv) reported more than one of the following primary outcome parameters: richness, abundance, evenness, α-diversity, or compositional dissimilarity (β-diversity). We did not include studies that were non-clinical (experimental and basic studies), observational, or retrospective. Study protocols, review articles, abstracts, editorials, and animal studies were excluded.

## Abbreviations

SA-AKI: sepsis-associated acute kidney injury
SCr: serum creatinine
ARC: augmented renal clearance
GFR: glomerular filtration rate
SIRS: systemic inflammatory response syndrome
LPS: lipopolysaccharide
CLP: cecal ligation and puncture
ICU: intensive care unit
SCFAs: short-chain fatty acids
I/R: renal ischemia–reperfusion
MLCK: myosin light chain kinase
Th17: T-helper 17
IS: indoxyl sulfate
RFR: renal function reserve
OR: odds ratio
CI: confidence interval
FMT: fecal microbiota transplantation
RBF: renal blood flow
SDD: selective digestive tract decontamination
CKD: chronic kidney disease
Stx2: Shiga toxin 2.
