# Supplementary material for: Contribution of gut microbiota toward renal function in sepsis
Source: Front Microbiol. 2022 Sep 6;13:985283. doi: 10.3389/fmicb.2022.985283 (PMC9486003; doi:10.3389/fmicb.2022.985283)
Supplement: Supplementary file 1 [file Table_1.DOCX]

| **PMID** | **Country** | **Year of experiment** | **Year of publication** | **Experimental group** | **Control group** | **Fecal sampling timeframes** | **Richness**  **(experimental group *vs.* control group)** | **Bacterial diversity** | **Gut dominant microbiota (phylum level)** | |
| --- | --- | --- | --- | --- | --- | --- | --- | --- | --- | --- |
|  |  |  |  |  |  |  |  |  | **experimental group** | **control group** |
| 34295779 [31] | China | 2016-2018 | 2021 | 25 patients with sepsis (3.29±3.07 years) | 15 age- and sex-matched healthy controls (2.80±1.77 years) | within 48 h after the diagnosis of sepsis | experimental group < control group | α-diversity: experimental group < control group | Proteobacteria | Firmicutes |
| 30261905 [36] | Japan | 2011-2016 | 2018 | 35 patients with sepsis receiving daily synbiotics [74 (64-82) year] | 37 patients with sepsis did not receive synbiotics [74 (64-81) year] | D0 | 6.8±0.4 *vs*. 8.1±0.4 (log10 cells/g of feces) | NA | Firmicutes (Clostridium leptum) | Firmicutes (Clostridium leptum) |
|  |  |  |  |  |  | W1 | 8.9±0.4 *vs*. 7.7±0.4 (log10 cells/g of feces) |  | Firmicutes (Clostridium leptum) | Firmicutes (Clostridium leptum) |
|  |  |  |  |  |  | W2 | 8.6±0.4 *vs.* 8.3±0.4 (log10 cells/g of feces) |  | Bacteroidetes (Bacteroides fragilis) | Bacteroidetes (Bifidobacterium) |
| 35173154 [37] | Netherlands | 2012-2016 | 2022 | 147 neonates with suspected early-onset neonatal sepsis receiving antibiotic therapy (49 per group): Group A (penicillin + gentamicin); Group B (co-amoxiclav + gentamicin); Group C (amoxicillin + cefotaxime) | 80 age-matched term-born healthy controls | D1 | NA | α-diversity: Group A > Controls > Group C > Group B | Group A&B&C: Proteobacteria (Escherichia coil) | Proteobacteria (Escherichia coil) |
|  |  |  |  |  |  | W1 |  | α-diversity: Controls > Group C > Group A > Group B | Group A: Firmicutes (Enterococcus faecium); Group B&C: Proteobacteria (Escherichia coil) | Bacteroidetes (Bifidobacterium) |
|  |  |  |  |  |  | M1 |  | α-diversity: Group A > Group C > Group B > Conrols | Group A&B&C: Bacteroidetes (Bifidobacterium) | Bacteroidetes (Bifidobacterium) |
|  |  |  |  |  |  | M4 |  | α-diversity: Group A > Group C > Controls > Group B | Group A&B&C: Bacteroidetes (Bifidobacterium) | Bacteroidetes (Bifidobacterium) |
|  |  |  |  |  |  | M12 |  | α-diversity: Group A > Group C > Controls > Group B | Group A&B&C: Bacteroidetes (Bifidobacterium) | Bacteroidetes (Bifidobacterium) |
| 30694100 [38] | Austria | 2015-2016 | 2019 | 9 patients with sepsis [54 (47, 60) years]: Group A (5 receiving probiotic); Group B (4 receiving placebo); | 21 healthy controls [58 (53, 63) years] | within 48 h after antibiotic therapy | experimental group < control group | α-diversity: experimental group < control group | Firmicutes | Bacteroidetes |
| 33898360[39] | China | 2018 | 2021 | 20 patients with sepsis: (10 per group): Group A (sepsis with confirmed pathogens infection) 3.40±1.43 years; Group B (sepsis without confirmed pathogens) 3.80±2.30 years | 20 healthy controls (6.35±2.0 years) | within 48 h after antibiotic therapy | Group B< Group A< control group | α-diversity: Group B < control group | Group A: Proteobacteria; Group B: Firmicutes | Firmicutes |
| 33607294[40] | China | 2016-2017 | 2020 | 47 ICU-enterotype I | 17 ICU-enterotype II | within 9 D after admission | ICU-E1 < ICU-E2 | α-diversity: ICU-E1 < ICU-E2 | Bacteroidetes | Firmicutes |
| 31465241[41] | China | 2019 | 2019 | 22 patients with sepsis | 32 healthy controls | within 48h after the diagnosis of sepsis | experimental group < control group | α-diversity: experimental group < control group | Firmicutes | Firmicutes |

**Table S1 Characteristics of the included studies**

Data are presented as the median (Q1; Q3) or mean ± standard deviation. H=hour, D = day, W = week, M=month.
