# Supplementary figures and images for: Contribution of gut microbiota toward renal function in sepsis
Source: Front Microbiol. 2022 Sep 6;13:985283. doi: 10.3389/fmicb.2022.985283 (PMC9486003; doi:10.3389/fmicb.2022.985283)

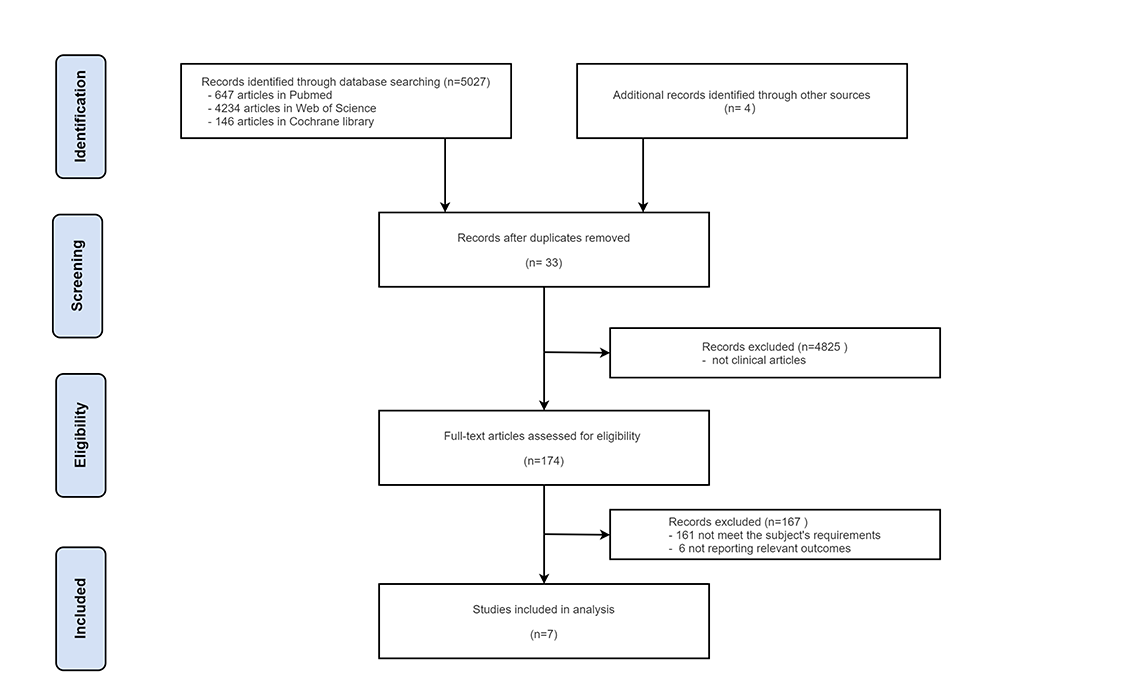

Supplement: Supplementary Figure 1 — Flow chart of literature selection process. Studies were included if they met the following criteria: (i) it was a randomized controlled trial targeting the gut microbiota of sepsis patients; (ii) all subjects were free from the original heart, brain, endocrine, and other basic diseases; (iii) species identification was performed by DNA sequencing of 16S rDNA; (iv) reported more than one of the following primary outcome parameters: richness, abundance, evenness, α-diversity, or compositional dissimilarity (β-diversity). We did not include studies that were non-clinical (experimental and basic studies), observational, or retrospective. Study protocols, review articles, abstracts, editorials, and animal studies were excluded. [file Image_1.TIFF]
